# Signal Regulatory Protein α Negatively Regulates β_2_ Integrin-Mediated Monocyte Adhesion, Transendothelial Migration and Phagocytosis

**DOI:** 10.1371/journal.pone.0003291

**Published:** 2008-09-29

**Authors:** Dan-Qing Liu, Li-Min Li, Ya-Lan Guo, Rui Bai, Chen Wang, Zhen Bian, Chen-Yu Zhang, Ke Zen

**Affiliations:** 1 Jiangsu Diabetes Research Center, State Key Laboratory of Pharmaceutical Biotechnology, School of Life Sciences, Nanjing University, Nanjing, Jiangsu, China; 2 Jiangsu CDC-Nanjing University Joint Institute for Virology, Nanjing, Jiangsu, China; Yale University School of Medicine, United States of America

## Abstract

**Background:**

Signal regulate protein α (SIRPα) is involved in many functional aspects of monocytes. Here we investigate the role of SIRPα in regulating β_2_ integrin-mediated monocyte adhesion, transendothelial migration (TEM) and phagocytosis.

**Methodology/Principal Findings:**

THP-1 monocytes/macropahges treated with advanced glycation end products (AGEs) resulted in a decrease of SIRPα expression but an increase of β_2_ integrin cell surface expression and β_2_ integrin-mediated adhesion to tumor necrosis factor-α (TNFα)–stimulated human microvascular endothelial cell (HMEC-1) monolayers. In contrast, SIRPα overexpression in THP-1 cells showed a significant less monocyte chemotactic protein-1 (MCP-1)–triggered cell surface expression of β_2_ integrins, in particular CD11b/CD18. SIRPα overexpression reduced β_2_ integrin-mediated firm adhesion of THP-1 cells to either TNFα–stimulated HMEC-1 monolayers or to immobilized intercellular adhesion molecule-1 (ICAM-1). SIRPα overexpression also reduced MCP-1–initiated migration of THP-1 cells across TNFα–stimulated HMEC-1 monolayers. Furthermore, β_2_ integrin-mediated THP-1 cell spreading and actin polymerization in response to MCP-1, and phagocytosis of bacteria were both inhibited by SIRPα overexpression.

**Conclusions/Significance:**

SIRPα negatively regulates β_2_ integrin-mediated monocyte adhesion, transendothelial migration and phagocytosis, thus may serve as a critical molecule in preventing excessive activation and accumulation of monocytes in the arterial wall during early stage of atherosclerosis.

## Introduction

Recruitment of monocytes from circulation to inflamed tissues plays a pivotal role in the initiation and progression of atherosclerosis [1], [2], [3]. After migrated to lesion region, monocytes are rapidly differentiated into macrophage which engulf lipids and form the fatty streak [4]. Although the mechanisms that govern the delivery of monocytes from circulation to inflammatory site are not fully understood, the process of monocyte diapedesis has been regarded as a multi-step event that is sequentially regulated by a panel of adhesion molecules and signaling pathways. E- and P-selectins are involved in the initial reversible adherence of monocytes to the endothelial cell monolayers [5]. The following firm adhesion is mediated by monocyte β_2_ integrins,including CD11a/CD18 and CD11b/CD18,that recognize vascular cell adhesion molecule-1(VCAM-1) and intercellular adhesion molecule-1 (ICAM-1) on endothelial cells [6]. Firm adhesion of monocytes requires activation of integrins, which can be triggered by agonist-induced activation of G protein–coupled chemokine receptors [7]. Monocytes express CC chemokine receptor 2 (CCR2), which binds monocyte chemoattractant protein-1 (MCP-1), leading to β_2_ integrin-mediated firm adhesion and subsequent transmigration of adhered monocytes through the vascular endothelium [8].

Recently signal regulatory protein α (SIRPα) (also designate as SHPS-1[9], p84[10], BIT[11], MFR[12], MyD-1[13], etc.) has been reported to serve as an important modulator for controlling leukocyte inflammatory responses [14], [15]. As an immunoglobulin superfamily member (IgSF), SIRPα is expressed mainly by myeloid. SIRPα has a long intracellular domain that contains four tyrosine residues to form two immunoreceptor tyrosine-based inhibition motifs (ITIMs) and this type of signaling structure is highly conserved between mice, rats and humans. Studies have suggested that binding of SIRPα with its extracellular ligand CD47 results in phosphorylations of SIRPα ITIMs, which in turn, leads to their association with SH2-domain-containing protein tyrosine phosphotases SHP-1 and SHP-2 [16], [17] to delivers signals that regulate a variety of cellular functions [14]. Ligation of SIRPα by antibody or CD47 recombinant inhibits many leukocyte functions, including phagocytosis[18], [19], tumour-necrosis factor production[20] and *in vitro* transmigration[21], [22]. Activation of SIRPα by arterial elastic laminae also inhibits monocyte adhesion[23]. Fibroblasts expressing a SIRPα mutant lacking ITIMs-containing cytoplasmic tail showed increased formation of focal adhesions and actin stress fibres in response to interaction with extracellular matrix, suggesting that SIRPα also plays a role in integrin-mediated cytoskeletal organization [24]. Negative regulatory role of SIRPα has also been found in tumor metastasis, survival, and cell transformation [25].

In the present study, to further explore the negative regulatory role of SIRPα in various functional aspects of monocytes, we examined the correlation between expression level of SIRPα in THP-1 cells and THP-1 cell transmigratory capacity. By overexpressing SIRPα in THP-1 cells, we also determined the alteration of β_2_ integrin expression and β_2_ integrins-mediated cellular functions of monocytes in response to chemoattractant stimulation.

## Materials and Methods

### Reagents and Antibodies

Recombinant human MCP-1 and TNFα, were purchased from PeproTech (Rocky Hill, NJ). AGEs-BSA (AGEs) was prepared according to a method previously described [26]. Briefly, 50 mg/ml bovine serum albimin (BSA) (Fraction V, sterile filtered, Sigma-Aldrich) was incubated with 0.6 M D-ribose or 0.5 M D-glucose and 0.3 M lysine in PBS containing 100 units/ml penicillin and streptomycin for 4 weeks. The unincorporated sugars were removed by dialysis against PBS. Polyclonal anti-human CD11b were generated against C-terminal peptide of CD11b [27]. Polyclonal anti-SIRPα antibody (SIRPα-ct) was obtained from Chemicon (Temecula, CA). Monoclonal anti-CD11b (OKM-1), and anti-CD11a (TIB-217), prepared in our laboratory from hybridoma, were obtained from American Type Culture Collection (ATCC) (Manassas, VA). Monoclonal anti-CD11c (S-HCL-3, IgG2b) and anti-CCR2 (clone 48607) were obtained from BD Biosciences (San Diego, CA).

### Cells

THP-1 cells (Chinese Cell Culture Center, Shanghai, China) were cultured and maintained as described [8]. In separated experiments, THP-1 cells were treated with AGEs-BSA (AGEs) at various concentrations overnight. Cells treated with BSA of the same concentration served as controls. Immortalized HMEC-1 was kindly provided by Dr. E.W. Ades (Centers for Disease Control and Prevention, Atlanta, GA)[28] and were grown in MCDB-131 (Invitrogen) supplemented with 10 ng/ml epidermal growth factor (Becton-Dickinson), 1 µg/ml hydrocortisone (Sigma-Aldrich) and 10–15% fetal bovine serum (Hyclone). HMEC-1 cells were seeded on collagen (Sigma-Aldrich)-coated tissue culture plates or permeable Transwell filters (5.0 µm pore size, 0.33 cm^2^ surface areas, Costar, NY).

### SIRPα Overexpression

Complete sequence of human SIRPα was amplified, inserted into the expression vector pcDNA3.1 (Invitrogen, Carlsbad, CA), and confirmed by DNA sequencing. The transfection of THP-1 cells was conducted via Lipofectmin 2000 (Invitrogen). Briefly, Lipofectmin-SIRPα pcDNA3.1 complex (250 µL) was added dropwise to 5×10^6^ THP-1 cells in 20 mL RPMI medium 1640 containing 2% heat-inactivated fetal bovine serum. After 24 h incubation, THP-1 cells were harvested and used for further analysis.

### Immunofluorescence, Confocal Microscopy and Flow Cytometry

Surface expressions of β_2_-integrins were detected using flow cytometry as described [8], [29]. The relative surface expression was estimated by subtracting the mean fluorescence intensity (MFI) of cells labeled with the nonspecific antibody from that of cells labeled with the antibodies detecting β_2_-integrins. All studies consisted of at least three independent experiments. Flow cytometry was performed and data were analyzed using CELLQUEST software (BD Biosciences). The polymerization of actin filaments in THP-1 cells, induced by pretreatment with 10 nM MCP-1 or 25 ng/mL TNFα for 30–60 minutes, was determined using rhodamine-conjugated phalloidin staining (Molecular Probes) according to the manufacturer's protocol. Briefly, cells treated with cytokines were fixed with 3.7% paraformaldehyde in PBS for 5 minutes, gently permeabilized with 0.1% Triton X-100, and blocked with 1% BSA in PBS for 30 min followed by rhodamine-conjugated phalloidin staining. In some experiments, cells were treated with cytochalasin D (Sigma-Aldrich) to inhibit actin polymerization [30]. Coverslips were mounted with antifade mounting medium (Molecular Probes). Images were captured and analyzed by a laser scanning confocal microscope equipped with an image processing system (Olympus Microsystems).

### Cell Adhesion Assays

Confluent HMEC-1 cell monolayers cultured on gelatin (Difco)–coated tissue culture plates or permeable Transwell filters. Monolayers were treated with 25 ng/mL TNFα for 6 h to induce VCAM-1 and ICAM-1 expression prior to the adhesion assay [31], [32]. In separate experiments, 24-well plates were coated for 2 h with 10 µg/mL human recombinant VCAM-1 or ICAM-1. THP-1 cells were briefly labeled with 2′,7′-bis-(2-carboxyethyl)-6-carboxyfluorescein acetoxymethyl ester (BCECF-AM, Molecular Probes)[27] and then suspended in RPMI 1640 medium containing 0.1% BSA and stimulated with 10 nM MCP-1 for 30 min to activate integrins [30]. In a subset of experiments, monocytes were pre-incubated with 50 µM dibutyl-cAMP (Bt2cAMP) for 30 min to inhibit chemokine-mediated integrin activation. Fluorescently labeled monocytes (∼2×10^5^ cells/well) were then added to HMEC-1 monolayers or 24-well plates coated with VCAM-1 or ICAM-1, and incubated for 30 min at 37°C. Nonadherent monocytes were removed by gentle washing with PBS and the bound cells were measured by a fluorescence plate reader at excitation/emission wavelengths of 485/535 nm (Millipore, Milford, MA)[27].

### Transendothelial Migration (TEM)

Migration of THP-1 cells across TNFα–pre-activated HMEC-1 monolayers was performed as previously described [30] with minor modification. Prior to migration assay, HMEC-1 monolayers cultured on gelatin (Difco)–coated transwell filters were treated with 25 ng/mL TNFα for 6 h. THP-1 cells (5.0×10^5^/per well) were added to the upper chamber of Transwell inserts containing 200 µl HBSS. 700 µl HBSS containing 10 nM MCP-1 was placed in the bottom chamber. After 90 min and 180 min incubation at 37°C under 5% CO_2_, cells that had transmigrated to the lower chamber were harvested in 1 ml PBS containing 0.1% BSA and labeled with phycoerythrin (PE)-conjugated anti-human CD14 antibody. 10^6^ FITC-conjugated standard beads (PharMingen, La Jolla, CA) were added to the cell suspension and the number of THP-1 cells was counted until 10,000 beads were counted by flow cytometry. All experiments were repeated as triplicated fashion in at least three independent studies.

### Phagocytosis of Fluorescein Conjugated Bacteria

THP-1 cells transfected with SIRPα or Mock vector were incubated for 3 h with 100 µl of fluorescein-conjugated *E. coli* K-12 bioparticles (Molecular Probes) [33]. The *E. coli* suspension was aspirated, and after three washes with Hank's balanced salt solution devoid of Ca^2+^ and Mg^2+^ (HBSS^−^), cell associated fluorescence was observed under microscopy or measured using a SpectraMax microtiter plate reader (Molecular Devices).

### Western Blot

THP-1 cells were solubilized in lysis buffer containing 1% Triton X-100 and a panel of protease inhibitors at 4°C. Pellet was removed after centrifuged at 13,000×*g* for 5 minutes. Supernatant was normalized for total protein, and loaded on 10% SDS-PAGE. After electrophoresis and transfer onto Hybond membranes, membranes were blocked with 5% non-fat milk. Antigens were detected using suitable primary antibodies followed by incubation with HRP-conjugated antibodies and ECL (Amersham) detection.

### Statistical Analysis

Data were analyzed by the Student *t* test; P values of <0.05 were regarded as significant differences (*, p<0.05; **, p<0.01).

## Results

### Downregulation of SIRPα in THP-1 cells is correlated with enhanced β_2_ integrin-mediated cell adhesion

**Figure 1:**
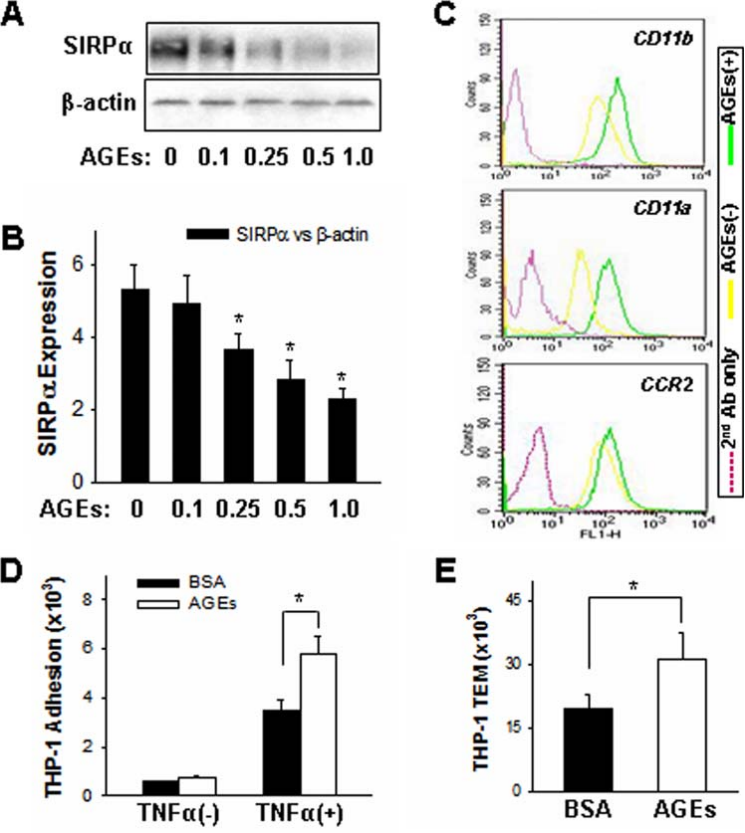
Down-regulation of SIRPα in THP-1 cells treated with BSA-AGEs is correlated to enhanced THP-1 cell surface expression of leukocyte β_2_ integrins and β_2_ integrins-mediated THP-1 cell inflammatory responses. In these experiments, THP-1 cells were treated with BSA-AGEs (AGEs) or BSA overnight at 37°C. A: SIRPα protein level in THP-1 cells; B: reduction of SIRPα protein level by AGEs in dose-dependent fashion; C: Cell surface expression level of β_2_ integrins in THP-1 cells; D and E: THP-1 cell adhesion to and migration across TNFα–pre-activated HMEC-1 monolayers, respectively. All data are mean±SD (*n* = 3) of three independent experiments.

It has been reported that advanced glycation end products (AGEs) are involved in tissue damage associated with diabetic complications and aging [34], [35]. Although the mechanism is still not clear, monocytes tend to be activated by AGEs and show an enhanced chemotaxis under such inflammatory conditions. As shown by Western blot analysis in Figure 1A, the expression of SIRPα in THP-1 cells was decreased after AGEs treatment. Served as controls, β-actin level was not altered. The down-regulation of SIRPα in AGEs–treated THP-1 cells is contrast to that of receptor for advanced glycation end products (RAGE) and junctional adhesion molecule-like protein (JAML), which expression levels are both increased after AGEs treatment (Zen et al, unpublished). Fig. 1B showed the quantitative analysis of SIRPα downregulation by AGEs in a dose-dependent fashion. Interestingly, AGEs–treated THP-1 cells showed a significant enhanced cell surface expression of β_2_ integrins, in particular CD11b/CD18, in response to the stimulation of MCP-1 (Fig. 1C). Also, compared to BSA–treated THP-1 cells, AGEs– treated THP-1 cells had a higher percentage of cell adhesion to TNFα–activated HMEC-1 monolayer (Fig. 1D). In response to MCP-1, AGEs–treated THP-1 cells also showed an increased transmigration across TNFα–activated HMEC-1 monolayers compared to THP-1 cells treated with BSA (Fig. 1E). Together, these results suggest that AGEs treatment can activate THP-1 cells and enhance cell chemotaxis.

### Overexpression of SIRPα in THP-1 cells inhibits MCP-1–induced cell surface expression of β2 integrins

**Figure 2:**
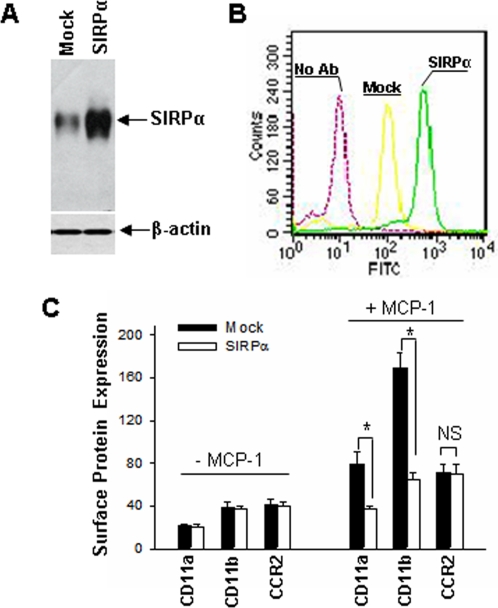
SIRPα overexpression in THP-1 cells and its effect on cell surface expression of β_2_ integrins and CCR2. THP-1 cells were transfected with the empty pcDNA3.1 vector (Mock) or the SIRPα-encoding pcDNA3.1 vector (SIRPα). A: SIRPα protein level in SIRPα– or mock– transfected THP-1 cells; B: Cell surface SIRPα expression in Mock– and SIRPα–transfected THP-1 cells; C: THP-1 cell surface expression of CD11a, CD11b, CD11c and CCR2. Note that SIRPα overexpression significantly suppressed MCP-1–induced up-regulation of THP-1 surface expression of CD11b and CD11a but not CCR2, and that SIRPα overexpression did not affect the basal level of β_2_ integrins. All data are mean±SD of three independent experiments.

To define the role of SIRPα in regulating monocyte inflammatory response, we characterized the alteration of β_2_ integrin expression and β_2_ integrin-mediated cell adhesion, migration and phagocytosis in THP-1 cells after significantly increase SIRPα expression level. As shown in Figure 2A, immunoblot analysis showed that the delivery of the pcDNA3.1 vector encoding human SIRPα into THP-1 cells profoundly enhanced SIRPα expression. Compared to mock–transfected THP-1 cells, SIRPα–transfected THP-1 cells also showed a significantly enhanced expression of SIRPα on cell surface, as indicated by flow cytometry (Fig. 2B).

Similar to circulating monocytes, THP-1 cells normally express β_2_ integrins [8], [36], [37] and their cell surface expression levels are rapidly up-regulated by chemoattractants during inflammatory response. We next determined the alteration of cell surface expression of CD11b/CD18 and CD11a/CD18 in SIRPα– or mock–transfected THP-1 cells. In these experiments, SIRPα– or mock–transfected THP-1 cells were treated with or without 10 nM MCP-1 for 30 min and then directly labeled with PE-conjugated mouse IgG specific for human CD11a, CD11b, and CC chemokine receptor 2 (CCR2), and surface expression was analyzed using flow cytometry. The results showed that SIRPα overexpression in THP-1 cells did not affect the basal level of β_2_ integrin expression on cell surface but significantly reduced the up-regulation of cell surface β_2_ integrin expression by MCP-1 (Fig. 2C).

### SIRPα overexpression affects CD11b/CD18-mediated THP-1 cell functions in response to MCP-1 stimulation

**Figure 3 pone-0003291-g003:**
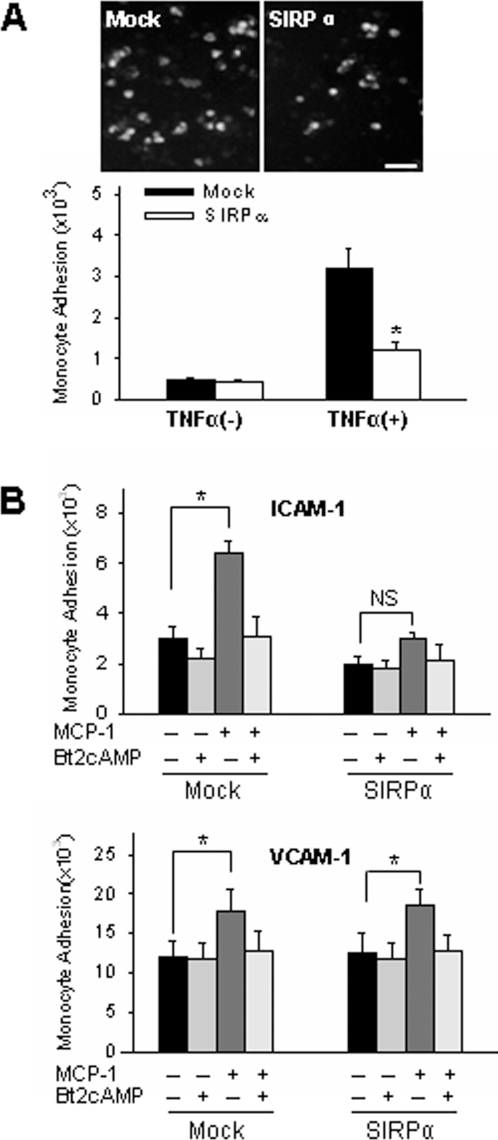
SIRPα overexpression in THP-1 cells decreases MCP-1–stimulated adhesion. A: Micrographs and histograms show THP-1 cells adhered to HMEC-1 monolayers. Bar = 20 µm. B: MCP-1–dependent adhesion of SIRPα– or mock–transfected THP1 monocytes to plates coated with ICAM-1 or VCAM-1. In separate experiments, monocytes were pretreated with 50 µM Bt2cAMP to determine background integrin-independent nonspecific adhesion. All data are mean±SD (*n* = 3) of three independent experiments.

Chemokines such as MCP-1 has been reported to trigger integrin–mediated firm adhesion and subsequent transmigration of monocytes [8]. As shown in Figure 3A, MCP-1–stimulated THP-1 cells showed a significant integrin-mediated firm adhesion to TNFα–activated HMEC-1 monolayers. However, this firm adhesion was largely reduced in THP-1 cells with SIRPα overexpression. Since TNFα–stimulated HMEC-1 monolayers express both VCAM-1 and ICAM-1, the specific ligands for β_1_ and β_2_ integrins, respectively, additional adhesion assays were performed using plates coated with human recombinant ICAM-1 or VCAM-1, respectively. To estimate background adhesion, control adhesion assays were performed using THP-1 cells pretreated with Bt2cAMP, a permeable analogue of cAMP that blocks integrin-dependent firm adhesion triggered by MCP-1 [30]. SIRPα–transfected THP-1 cells did not show MCP-1–induced firm adhesion to plates coated with ICAM-1, while adhesion to plates coated with VCAM-1 was intact (Fig. 3B).

**Figure 4 pone-0003291-g004:**
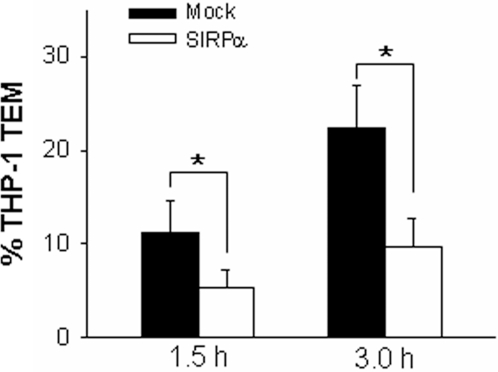
SIRPα overexpression reduces the MCP-1–induced migration of THP-1 cells across HMEC-1 monolayers pre-activated with 25 ng/mL TNFα. All data are mean±SD (*n* = 3) of three independent experiments.

Previous studies have reported that chemokine-mediated activation of β_2_ integrins was essential for THP-1 cell adhesion and subsequent transmigration through endothelial monolayers [7], [8], [38]. Therefore, the effect of SIRPα overexpression on transendothelial migration (TEM) of THP-1 cells was examined by transmigration assay. In mock-transfected THP-1 cells, MCP-1 triggered strong THP-1 cell migration across HMEC-1 monolayers. As shown in Figure 4, more than 20% of total applied THP-1 cells were migrated across TNFα–activated HMEC-1 monolayers after 3 h incubation. In contrast, MCP-1–triggered transmigration of THP-1 cells that were overexpressed with SIRPα was strongly reduced (Fig. 4). In both mock-transfected and SIRPα overexpressed THP-1 cells, spontaneous migration of THP-1 cells in the absence of MCP-1 was minimal (data not shown).

**Figure 5 pone-0003291-g005:**
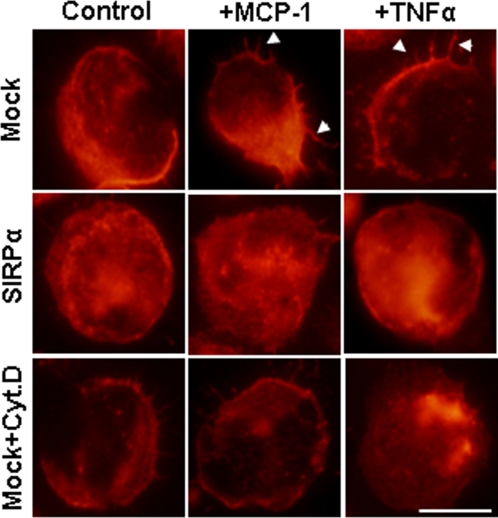
TNFα and MCP-1–induced cell spreading and actin polymerization in THP-1 cells. SIRPα– or mock–transfected THP-1 cells were stimulated with TNFα or MCP-1 for 30 minutes, fixed, and labeled with rhodamine-conjugated phalloidin to visualize actin filaments. As a negative control, mock–transfected THP-1 cells were pretreated with cytochalasin D (Mock+Cyt. D). Bar = 20 µm.

Leukocyte β_2_ integrin–mediated cell firm adhesion initiates cell shape changes and spreading of monocytes, events that must occur for subsequent cellular locomotion and transmigration. Next we investigated the effect of SIRPα overexpression on TNFα– and MCP-1–stimulated actin polymerization and cell spreading in THP-1 cells. SIRPα– or mock– transfected THP-1 cells were stimulated with TNFα or MCP-1 for 30 minutes, then fixed, and labeled with rhodamine-conjugated phalloidin to visualize actin filaments. As a control, mock–transfected THP-1 cells were pretreated with cytochalasin D to inhibit actin polymerization [39]. Labeled monocytes were mounted on coverslips and images were obtained using confocal microscopy. As shown in Figure 5, confocal microscope images showed that mock– transfected THP-1 cells exposed to TNFα or MCP-1 underwent morphological changes resulting in multiple pseudopods (arrowheads) with abundant actin filaments, and that this process was inhibited by cytochalasin D. In contrast, significantly less TNFα– or MCP-1–stimulated actin polymerization and cell spreading had occurred in THP-1 cells with SIRPα overexpression (Fig. 5).

**Figure 6:**
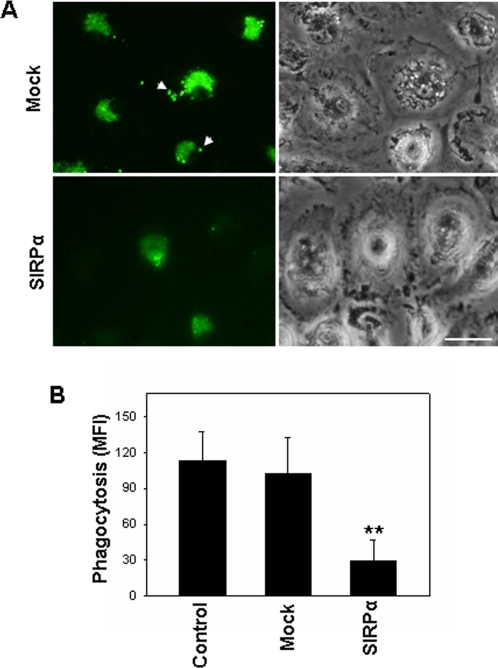
SIRPα overexpression in THP-1 cells impairs cell phagocytic function. Fluorescently labeled bacteria were incubated with SIRPα– or mock–transfected THP-1 cells for 3 h. A: Images of phagocytosis of bacteria by THP-1 cells; B: Quantitative analysis of bacteria phagocytosis by THP-1 cells. Bar = 20 µm. All data are mean±SD (*n* = 4) of four independent experiments.

The phagocytic function of THP-1 cells is also dependent on β_2_ integrins [40], [41], [42]. Next we examined the effect of SIRPα overexpression on the capacity of THP-1 cells to engulf fluorescein–labeled *E. coli* K12 bioparticles. As shown by confocal images in Figure 6A, the mock-transfected THP-1 cells showed a significant phagocytosis of fluorescein–conjugated bacteria particles after 3 h incubation (Fig. 6A, arrows), while in SIRPα–transfected THP-1 cells, uptake of fluorescein–conjugated bacteria particles was strongly reduced. The quantitative analysis of uptaking fluorescein–conjugated bacteria particles by THP-1 cells was shown in Figure 6B. Taken together, these results clearly show that SIRPα overexpression in THP-1 cells reduces various inflammatory responses mediated by leukocyte β_2_ integrins.

## Discussion

Recent studies have demonstrated that SIRPα is involved in regulating various inflammatory responses of leukocytes, in particular leukocyte chemotaxis and phagocytosis. By studying the leukocyte β_2_ integrin-mediated functional changes in THP-1 cells after downregulation or overexpression of SIRPα level, we show that SIRPα negatively regulates β_2_ integrin-mediated THP-1 cell inflammatory responses, such as adhesion, transendothelial migration and phagocytosis.

### Correlation between SIRPα protein level and CD11b/CD18-mediated cellular functions in THP-1 cells

Ligation of SIRPα with its extracellular ligand CD47 results in phosphorylations of SIRPα ITIMs, which in turn, leads to their association with SH2-domain-containing protein tyrosine phosphotases SHP-1 and SHP-2 [16], [17] to delivers signals that regulate a variety of cellular functions [14]. Binding of SIRPα by antibody or CD47 recombinant inhibits many leukocyte functions, including phagocytosis [18], [19], tumour-necrosis factor production [20] and *in vitro* transmigration [21], [22]. Activation of SIRPα by arterial elastic laminae also inhibits monocyte adhesion [23]. Fibroblasts expressing a SIRPα mutant lacking ITIMs-containing cytoplasmic tail showed increased formation of focal adhesions and actin stress fibres in response to interaction with extracellular matrix, suggesting that SIRPα is also involved in mediating outside-in signal transduction during cell-matrix interaction. Using THP-1 cell as model cell line, here we show that SIRPα protein level is downregulated by AGEs treatment, which is also correlated to an enhanced cell surface expression of β_2_ integrins and β_2_ integrins-mediated cell adhesion (Fig. 1). The finding of SIRPα reduction in AGEs-treated THP-1 cells is supported by a recent report that mouse macrophages have lower SIRPα expression level following LPS stimulation [43]. The correlation between SIRPα expression level and chemoattractant-induced cell surface upregulation of β_2_ integrins and β_2_ integrins-mediated THP-1 cell inflammatory responses is further characterized in THP-1 cells overexpressed with SIRPα (Fig. 2–6). The results not only confirm the inhibitory function of SIRPα on THP-1 inflammatory responses, but also indicated that the role of SIRPα in THP-1 cells is through affecting the functions of β_2_ integrins, particularly CD11b/CD18. It is worthy to note that overexpression of SIRPα does not alter the basal level of β_2_ integrin expression but the upregulation of β_2_ integrins by MCP-1 stimulation, suggesting that SIRPα is one of essential molecules along the signal pathways that may regulate the synthesis, transportation and translocation process of β_2_ integrins. Moreover, if AGEs and other inflammatory factors can affect β_2_ integrin expression and function through down-regulating SIRPα, it might be reasonable to conclude that SIRPα can mediate an inside-out signal in regulating β_2_ integrin function.

### SIRPα as a negative regulator in monocyte recruitment during inflammation

The expression of β_2_ integrins and adhesion molecules in monocytes is regulated by chemokines such as MCP-1, SDF-1 alpha and RANTES [32], [44], [45], [46]. The positive correlation between CD11b expression in circulating monocytes and the degree of monocyte infiltration into the proatherogenic vascular wall has been well-documented [8], [47], [48]. The increased expression of monocyte CD11b under pro-inflammatory conditions enhanced MCP-1–mediated chemotaxis *in vitro* [8], induced excess monocyte adhesion to vascular endothelium, and increased formation of neointima and atherosclerotic plaques [48]. Although SIRPα overexpression did not affect surface expression of CCR2, the receptor for MCP-1, it resulted in a profound reduction of MCP-1–mediated upregulation of THP-1 cell cell surface β_2_ integrins and THP-1 cell TEM. In addition to reduction of CD11b and other β_2_ integrins, our study has also demonstrated that overexpressing SIRPα in THP-1 cells display less cell spreading and actin polymerization in response to chemokine stimulation. The mechanism by which SIRPα modulates chemokine-induced cell spreading and actin polymerization is unknown although several possibilities exist: a) directly activates protein phosphatase and initiates signal pathways that attenuate filament actin polymerization and cell spreading, and b) binding to integrin-associated protein CD47 and modulating the integrin functions. Since SIRPα is a cellular ligand of CD47, which can augment the functions of integrins of the β_1_, β_2_ and β_3_ families via initiating heterotrimeric Gi protein signaling [49], thus modulating a range of cell activities including cell motility and adhesion, and leukocyte adhesion, migration and phagocytosis. Indeed, phagocytosis of bacteria by THP-1 cells, an event that is largely dependent on β_2_ integrin and actin polymerization, was significantly reduced by overexpression of SIRPα. This result was in agreement with the previous finding that SIRPα contributes to down-regulating the macrophage phagocytic response [18]. In summary, the present study demonstrates for the first time that SIRPα overexpression potently inhibits the various inflammatory responses of THP-1 monocytes/macrophages mediated by β_2_ integrins. The induction of SIRPα expression in THP-1 cells led to a reduction of chemokine-induced cell surface expression of β_2_ integrins, which eventually resulted in less cell adhesion, cellular spreading, cell transmigration and phagocytosis. This observation suggests that SIRPα may function to decrease transendothelial migration of monocytes or other circulating leukocytes, reduce the burden of inflammatory cells in atheroma, and ultimately decrease plaque mass under atherogenic conditions. Since migration of monocytes across blood vessel lining endothelial monolayers is a key component during early stage of atherosclerosis, such an outcome would indicate that SIRPα overexpression in monocytes or macrophages has an anti-atherogenic effect and that SIRPα is a potential target in therapeutical implications.
